# Optimization and Clinical Evaluation of an Automated Commercial Analyte-Specific Reagent Assay for *Mycoplasmoides genitalium* Macrolide Resistance Detection in Primary Clinical Specimens

**DOI:** 10.1128/jcm.00335-23

**Published:** 2023-06-21

**Authors:** Erik Munson, Amanda Zapp, Josephine Moore, Stephen C. Lavey, Hunter Russell, Kimber L. Munson, Irene Martin

**Affiliations:** a Department of Medical Laboratory Science, Marquette University, Milwaukee, Wisconsin, USA; b Wisconsin Clinical Laboratory Network Laboratory Technical Advisory Group, Madison, Wisconsin, USA; c Carleton College, Northfield, Minnesota, USA; d Ascension Wisconsin, Milwaukee, Wisconsin, USA; e National Microbiology Laboratory Public Health Agency of Canada, Winnipeg, Canada; Medical College of Wisconsin

**Keywords:** *Mycoplasmoides genitalium*, automation, commercial assay, macrolide resistance

## Abstract

With improvement in laboratory diagnosis of *Mycoplasmoides genitalium* infection through molecular diagnostics, macrolide resistance determination within M. genitalium-positive patients is necessary. In this study, we report baseline parameters for an analyte-specific reagent (ASR) macrolide resistance real-time reverse transcriptase PCR on an open access analyzer and evaluated detection of macrolide resistance-mediated mutation (MRM) within 23S rRNA in a clinical specimen set. Initial use of 1.2 μM M. genitalium primer and 0.8 μM M. genitalium detection probe concentrations yielded an 80% false-positive detection rate when challenged with 10,000 copies of wild-type RNA. Optimization experiments showed that lowering primer/detection probe and MgCl_2_ concentrations minimized these false-detections of wild-type 23S rRNA, while higher levels of KCl increased rates of MRM detection with concomitant lower cycle threshold values and higher fluorescence emission. Lower limit of A2058G mutation detection was 5000 copies/mL (180 copies/reaction; 20/20 detections). Utilization of a baseline correction slope limit of 250 units further mitigated false-detection from wild-type 23S rRNA at challenges up to 3.3 billion copies/mL. MRM was detected in 583/866 (67.3%) clinical specimens initially positive for M. genitalium by commercial transcription-mediated amplification. These data included 392/564 detections (69.5%) from M. genitalium-positive swab specimens and 191/302 (63.2%) from M. genitalium-positive-positive first-void urine specimens (*P = *0.06). Overall resistance detection rates did not vary by gender (*P = *0.76). Specificity of the M. genitalium macrolide resistance ASR was 100% (141 urogenital determinations). MRM detection by the ASR was confirmed at a concordance rate of 90.9% by Sanger sequencing of a clinical specimen subset.

## INTRODUCTION

Molecular diagnostics constitutes an emerging reference standard for laboratory detection of the sexually-transmitted infection (STI) agent *Mycoplasmoides genitalium* (1). To an extent, these assays from both performance and logistic perspectives have usurped previous diagnostic modalities, such as serology (due to M. genitalium antigenic variation and potential cross-reactivity with other members of order *Mollicutes* [2–7]) and culture (due to the fastidious nature of M. genitalium and/or extended time to reportable result [8, 9]).

Three commercial molecular diagnostic assays have been granted United States Food and Drug Administration (FDA) clearance for amplification/detection of M. genitalium DNA (10, 11), RNA (12), and rRNA (13, 14) from selected primary clinical specimens, such as first-void urine (both genders), self-collected vaginal swabs, provider-collected vaginal swabs, endocervical swabs, and self-collected meatal swabs. In some locales, studies have demonstrated equivalent or increased detection of M. genitalium-specific nucleic acid compared to that of Chlamydia trachomatis (15, 16). Moreover, off-label performance of commercial M. genitalium nucleic acid amplification testing on self-collected male rectal swab specimens as a laboratory-modified test has identified additional carriers of this STI agent (17, 18).

Point mutation-conferred macrolide resistance, as detected within M. genitalium 23S rRNA base positions 2058 and 2059 (Escherichia coli numbering), has been associated with azithromycin treatment failure (19, 20). Global surveys of primary clinical specimens with detectable M. genitalium nucleic acid have revealed increased frequency of macrolide resistance determinants, including data from North America reporting a 45% resistance rate in female urogenital specimens (21), a 64% rate in male urethral specimens from a United States multicenter study (range 60 to 76% by location) (22), and an approximate 70% rate in heterosexual couple urogenital, pharyngeal, and rectal specimens (23). As a result, the 2021 revised United States Centers for Disease Control and Prevention STI treatment guidelines (24) for M. genitalium are predicated based on availability of macrolide resistance detection assays, following a positive molecular diagnostic test result. However, commercial M. genitalium macrolide resistance detection assays are neither FDA-cleared nor widely available in the United States. In this report, we provide optimized parameters for an analyte-specific reagent macrolide resistance detection assay on an automated open access analyzer (heretofore referred to as Mgen MacR ASR) and provide data relative to macrolide resistance detection in a large primary clinical specimen collection.

## MATERIALS AND METHODS

### Nucleic acid reagents.

*In vitro* transcript (IVT) RNA corresponding to M. genitalium wild-type 23S rRNA at a stock concentration of 5 × 10^10^ copies/mL and IVT RNA constructs representing M. genitalium macrolide resistance-mediated mutation (MRM) A2058C, A2058G, A2058T, A2059C, and A2059G at concentrations of 10^7^ copies/mL were used as negative and positive controls, respectively. Stock IVT RNAs (all provided by Hologic, Incorporated) were diluted in sterile, nuclease-free water to working concentrations, and then frozen at −70°C. Cartridges containing lyophilized nucleotide bases, reverse transcriptase, and DNA polymerase were provided by Hologic for input into the Fusion system.

### Primer probe reconstitution preparation.

Mixtures of M. genitalium MRM primer sequences, M. genitalium MRM detection probe sequences, internal control primer sequences, internal control detection probe sequences, MgCl_2_, and KCl (collectively referred to as primer probe reconstitution [PPR]), with adjustment to specific molarity by nuclease-free water and additive 10 millimolar (mM) Tris buffer, were prepared for reverse transcriptase PCR optimization in 2.0-mL DNase-, RNase-free microcentrifuge vials (Simport Scientific, Incorporated). All PPR reagents were provided by Hologic. Stoichiometry of PPR reagents was determined by the user.

The M. genitalium primer sequence reagent was a proprietary mixture of primers specific to MRM (A2058C, A2058G, A2058T, A2059C, and A2059G), with corresponding detection probes labeled with a FAM channel fluorescence tag (excitation 460 nm/detection 517 nm). Proprietary internal control primer and detection probe pairs were labeled with a Quasar 705 channel fluorescence tag (excitation 690 nm/detection 720 nm). Upon reagent addition, vials were vortexed for 15 s, then pulse centrifuged. Following mineral oil overlay, contents were again pulse centrifuged before loading onto the Fusion system.

### Panther Fusion system.

For Mgen MacR ASR optimization experiments, known concentrations of wild-type 23S rRNA or MRM RNA were delivered to Aptima Unisex Swab Specimen Collection Kit tubes containing 2.9 mL specimen transport medium lysis buffer (Hologic). Direct tube sampling and initial processing occurred on the Panther analyzer, facilitated by Fusion enhancer reagent-S, magnetic bead-based Fusion capture reagent-S, and Fusion internal control template-S (Hologic). Following approximately 80 min of automated extraction, nucleic acids were automatically transferred to the Fusion analyzer for real-time reverse transcriptase PCR.

Amplification parameters included an initial reverse transcription step of 46°C for 8 min, 95°C for 2 min, followed by 45 cycles of 95°C for 5 sec and 60°C for 22 sec. These parameters, along with FAM and Quasar 705 fluorescence channel thresholds of 1000 units, were programmed into off-line MyAccess computer software (Hologic). MyAccess was designed to create open access protocols with user-defined settings that are loaded onto the Panther Fusion system.

### Primary clinical specimens.

Remnant primary clinical swab and first-void urine specimens from both genders, previously assayed by Aptima Combo 2 Assay (Hologic) for Chlamydia trachomatis 23S rRNA and Neisseria gonorrhoeae 16S rRNA, by Aptima Trichomonas vaginalis Assay (Hologic) for Trichomonas vaginalis 16S rRNA, and Aptima Mycoplasma genitalium Assay (Hologic) for M. genitalium 16S rRNA, were gathered for clinical verification. Within this collection, a convenience sample focused on 868 specimens that tested positive for M. genitalium-specific RNA.

### Pretreatment experiments for assessment of Mgen MacR ASR and comparison to untreated specimens.

Primary clinical specimens were pretreated prior to direct tube sampling to align with an extraction protocol recommended by Hologic (25). For swab specimens, 300 μL aliquots of residual material were delivered to clean, conical bottom specimen tubes (Phillips Plastics Corporation) to which a 300 μL aliquot of specimen transport medium/proprietary open access diluent additive (in a 100:1 volume/volume ratio) was dispensed (25). For first-void urine specimens, 600 μL aliquots of residual urine from Aptima Urine Specimen Collection Kit transport tubes (Hologic) were added to clean, conical bottom specimen tubes to which 3.75 μL of the proprietary open access diluent additive was dispensed (25). When residual specimen volume allowed following the pretreatment protocol, the remainder of the specimen was transferred to a clean, conical bottom specimen tube for Mgen MacR ASR analysis of untreated specimens. All specimen tubes were sealed with Aptima penetrable caps (Hologic) prior to loading onto the Panther Fusion system.

### Specificity assessments.

A total of 141 residual primary clinical specimens, yielding negative results from the Aptima M. genitalium assay, were processed to assess specificity of the Mgen MacR ASR. Furthermore, clinically-significant isolates of Staphylococcus aureus (26) and Streptococcus pneumoniae (27) exhibiting variable antimicrobial susceptibility phenotypes that included macrolide resistance (all with erythromycin MIC values > 16 μg/mL) were cultivated from surveillance collections and adjusted to a concentration of 1.5 × 10^8^ organisms/mL. Between 3 × 10^7^ and 5 × 10^7^ bacteria were delivered to Aptima Urine Specimen Collection Kit or Aptima Multitest Swab Specimen Collection Kit tubes containing specimen transport medium lysis buffer (Hologic) for performance of Mgen MacR ASR.

Limited proprietary data shared by the manufacturer indicated that primer and detection probe sequences inherent to Mgen MacR ASR did not demonstrate *in silico* binding affinity for 44 bacterial, fungal, and/or parasitic agents, including the *Mollicutes* bacteria *Metamycoplama hominis*, *Mycoplasmoides pneumoniae*, Ureaplasma parvum, and Ureaplasma urealyticum (D. Getman, A. Nenninger, J. Zowalki, personal communication).

### Inhibitory/interfering substances.

A panel of clinical urine and swab specimens from which MRM was previously detected by Mgen MacR ASR was assembled. In 1 set of experiments, 1 swab tip-full of commercial gynecologic lubricant was added to the bottom of clean, conical bottom specimen tubes, followed by 300 μL or 600 μL aliquots of swab and urine specimens from the panel, respectively, and by pretreatment volumes of open access diluent additive described in a previous section. In a second set of experiments, 1 small spatula tip-full of commercial talcum powder (average mass, 10.86 mg per tube) was added to the bottom of clean, conical bottom specimen tubes, followed by 300 μL or 600 μL aliquots of swab and urine specimens from the panel, respectively, and by pretreatment volumes of open access diluent additive. In a third set of experiments, human whole blood or seminal fluid was dispensed into the bottom of clean, conical bottom specimen tubes, followed by appropriate volumes of urine or swab specimens from the panel to create a 10% whole blood or seminal fluid/total volume ratio. To these suspensions were added appropriate pretreatment volumes of open access diluent additive.

Finally, to further assess the influence of potential endogenous urine and fecal inhibitory agents on primary clinical specimens possessing MRM (via Mgen MacR ASR), residual contents of 10 primary clinical rectal swab specimens (yielding negative results by Mgen MacR ASR) were pooled, as were residual contents of 5 primary clinical urine specimens (non-detectable MRM). Subsequently, 120 μL aliquots of primary urine specimens from the panel were dispensed into 480 μL aliquots of pooled rectal swab contents. In analogous fashion, 60 μL aliquots of primary swab specimens from the panel were dispensed into 240 μL aliquots of pooled primary urine contents. To these suspensions were added appropriate pretreatment volumes of open access diluent additive.

### Nucleic acid sequencing.

For confirmation of MRM detection status in the context of Mgen MacR ASR testing, a subset of untreated specimens (48 first-void urine specimens; 47 swab specimens) was extracted on the Roche MagNA Pure platform per manufacturer instructions. Sanger sequencing was used to identify mutations associated with macrolide resistance at region V of the 23S rRNA subunit (28).

### Data analysis.

Absence of internal control amplification was interpreted as a non-valid result. The Fusion analyzer generated both qualitative interpretation of Mgen MacR ASR results (macrolide resistance analyte; internal control analyte) and quantitative cycle threshold values (C*_T_*) when present. All data from batched runs were downloaded to a removable USB drive, and then analyzed by MyAccess software for both Relative Fluorescence Unit data and visualization of (sigmoidal) fluorescence output kinetics. The application was also used to view open access amplification curves and for analysis of processed data utilizing user-defined results interpretation parameters. One proprietary component of the software was a baseline correction algorithm. Baseline correction adjusted kinetic curves to reduce assay-specific ramping of fluorescence during the baseline portion of a PCR amplification curve. Baseline correction slope limit was defined as the maximum change in cycle-to-cycle fluorescence that the algorithm determines to be baseline ramping and not true amplification (J. Zowalki, personal communication). During initial experimentation, a default baseline correction slope limit was established at 50 units.

The significance test of proportions was used to determine if comparative proportion or frequency value differences were significant. Differences in C*_T_* or Relative Fluorescence Unit values were analyzed by the *t* test for independent samples. An *a priori* decision was made to examine statistical significance using a two-tailed test with an alpha level of 0.05.

## RESULTS

### Mitigation of preliminary false-detections of macrolide resistance in M. genitalium wild-type RNA template.

In initial experiments, M. genitalium wild-type RNA template (serving as a negative control) was introduced to Aptima specimen tubes at a concentration of 10^4^ copies/mL. The mock specimens were subsequently interrogated with a PPR solution containing separate 1.2 μM primer sequences targeting M. genitalium macrolide resistance determinants and internal control, with separate 0.8 μM detection probe sequences for the amplicons, with 60 mM KCl, and 3 mM MgCl_2_. Four of five (80%) tests yielded detectable signal. Within these false-detections, mean and median C*_T_* were 40.9 and 40.7, respectively. When primer/detection probe sequence concentrations were reduced to 0.6 μM and 0.4 μM, respectively, the false-detection rate within a subset of 40 interrogations of 10^4^ copies/mL of template was reduced to 15% at this point of experimentation (data not illustrated).

Wild-type M. genitalium RNA was further used as target sequences in experiments designed to titrate MgCl_2_ concentration within Mgen MacR ASR. When PPR containing the reduced primer/detection probe concentrations and 3 mM MgCl_2_ was added to 10^4^ copies/mL of template, a 19.0% (4/21) false-detection rate was observed (Table 1). This false-detection rate increased to 68.2% (15/22) when 10^5^ copies/mL of template RNA were introduced. In contrast, when MgCl_2_ concentration was reduced to 2 mM, false-detection rates were ≤ 10.5%, regardless of template RNA concentration. Table 1 data further suggest that false-detection rates derived from assessment of M. genitalium wild-type RNA were more dependent on MgCl_2_ concentration than KCl concentration.

**TABLE 1:** Detection rates of M. genitalium wild-type template 23S rRNA in the presence of 0.6 μM primer sequence and 0.4 μM detection probe sequence in the Mgen MacR ASR PPR, as a function of input target nucleic acid and additive KCl and MgCl_2_ concentrations

Wild-type M. genitalium template 23S rRNA present in Aptima specimen tube	Frequency of signal detection (%) stratified by KCl and MgCl_2_ concentration		
	KCl concentration	MgCl_2_ concentration	
		2 mM MgCl_2_	3 mM MgCl_2_
10^4^ copies/mL	40 mM	0/6 (0.0)	0/5 (0.0)
	60 mM	1/7 (14.3)	3/10 (30.0)
	80 mM	1/6 (16.7)	1/6 (16.7)
	Any KCl concentration	2/19 (10.5)	4/21 (19.0)

10^5^ copies/mL	40 mM	1/6 (16.7)	7/8 (87.5)
	60 mM	0/9 (0.0)	1/6 (16.7)
	80 mM	0/6 (0.0)	7/8 (87.5)
	Any KCl concentration	1/21 (4.8)	15/22 (68.2)

### Further optimization of PPR using M. genitalium MRM RNA constructs.

Upon reduction of false-detection rates from wild-type 23S rRNA template, KCl concentration was further optimized using M. genitalium MRM target. At 0.6 μM primer concentration and 0.4 μM detection probe concentration, challenge with 10^4^ copies/mL of A2058C, A2058T, A2059C, and A2059G RNA constructs resulted in signal detection in 100% of interrogations (total *n* = 154). Signal was detected in 97.5% of A2058G assessments at 10^4^ copies/mL. Analogous experiments with 10^5^ copies/mL of target RNA resulted in a detection rate of 100% (total *n* = 200 determinations).

Additional studies optimized cofactor concentrations for detection of all 5 MRM. Optimization of conditions relative to A2058G detection are presented herein, as this mutation is detected with high frequency in several demographics (29–34). When differing KCl concentrations were incorporated in PPR solutions containing 0.6 μM primer sequence, 0.4 μM detection probe sequence, and 2 mM MgCl_2_, mean and median Relative Fluorescence Unit values were increased at higher KCl concentrations when compared to 40 mM KCl, with concomitant decrease of mean and median C*_T_* (Table 2). This association was statistically significant when higher target RNA burden was interrogated (*P ≤ *0.016 for mean Relative Fluorescence Units; *P ≤ *0.023 for C*_T_*).

**TABLE 2:** Fluoresence data output from Mgen MacR ASR PPR consisting of 0.6 μM primer sequence, 0.4 μM detection probe, and 2 mM MgCl_2_ concentrations, stratified by KCl concentration and copies/mL of challenge A2058G M. genitalium RNA

KCl concentration	10^4^ Copies/mL A2058G target RNA present in Aptima specimen tube				10^5^ Copies/mL A2058G target RNA present in Aptima Specimen Tube			
	Mean C*_T_*	Median C*_T_*	Mean relative fluorescence units	Median relative fluorescence units	Mean C*_T_*	Median C*_T_*	Mean relative fluorescence units	Median relative fluorescence units
40 mM	37.92^*a*^	37.75	5699^*b*^	5842	34.97^*c*^	35.15	7756^*d*^	7583
60 mM	37.84	37.70	6180	6147	34.30	34.30	9366	9347
80 mM	37.63	37.70	6482	6640	34.28	34.25	10082	10263

a *P* ≥ 0.48 for all mean C*_T_* comparisons.

b *P* ≥ 0.23 for all mean Relative Fluorescence Units comparisons.

c *P* ≤ 0.023 for mean C*_T_* comparisons to 40 mM KCl; *P* = 0.91 for 60 mM KCl versus 80 mM KCl.

d *P* ≤ 0.016 for mean Relative Fluorescence Units comparisons to 40 mM KCl; *P* = 0.28 for 60 mM KCl versus 80 mM KCl.

**FIG 1:**
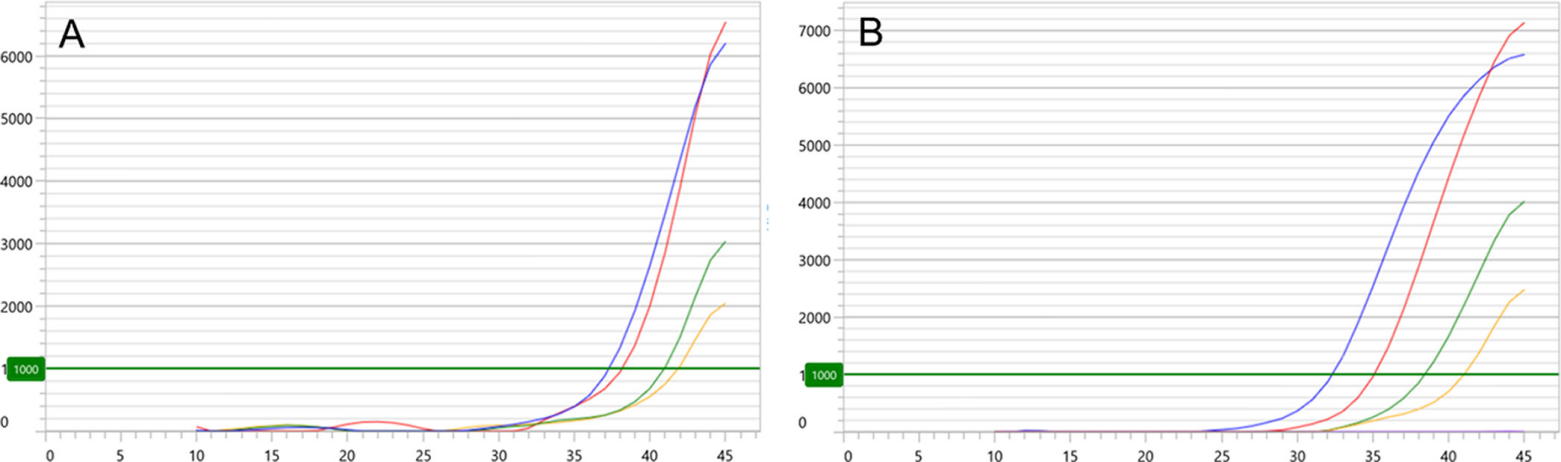
Mgen MacR ASR kinetic fluorescence output of representative limit of detection experiments interrogating (A) A2058G mutation target RNA at 10^4^ copies/mL in Aptima specimen tube (blue line), 5 × 10^3^ copies/mL (red), 10^3^ copies/mL (green), and 5 × 10^2^ copies/mL (orange); and, (B) clinical first-void urine specimen diluted 1:10 (blue line), 1:100 (red), 1:1000 (green), 1:10,000 (orange), and 1:100,000 (purple). Interrogations were made with PPR containing 0.6 μM primer sequence, 0.4 μM detection probe sequence, 2 mM MgCl_2_, and 60 mM KCl. *y* axis represents Relative Fluorescence Units (threshold set at 1000); *x* axis represents cycles of reverse transcriptase PCR.

In the limit of detection experiments, input A2058G mutation target RNA was interrogated by PPR containing 0.6 μM primer sequence, 0.4 μM detection probe sequence, 2 mM MgCl_2_, and 60 mM KCl. Input target burdens of 10^4^ copies/mL and 5 × 10^3^ copies/mL (360 copies/reaction and 180 copies/reaction, respectively) were detected in 100% of determinations (Table 3). In contrast, target burdens of 10^3^ and 5 × 10^2^ copies/mL (36 and 18 copies/reaction, respectively) were detected in 60% and 10% of determinations, respectively. One detectable output from assessment of 5 × 10^2^ copies/mL of A2058G target yielded C*_T_* and Relative Fluorescent Unit values of 41.9 (Fig. 1) and 2048, respectively. PCR efficiency was calculated at 106.9% (5 replicates at 3 target concentrations; data not illustrated).

**TABLE 3:** Limit of detection assessments for A2058G target RNA interrogated by Mgen MacR ASR PPR consisting of 0.6 μM primer sequence, 0.4 μM detection probe sequence, 2.0 mM MgCl_2_, and 60 mM KCl

A2058G target RNA present in Aptima specimen tube	Number of determinations	Number of detections	Mean C*_T_* value within detections	Median C*_T_* value within detections	Mean relative fluorescence unit value within detections	Median relative fluorescence unit value within detections
10^4^ copies/mL	20	20	38.22	38.15	5219	5345
5 × 10^3^ copies/mL	20	20	39.35	39.15	5240	5175
10^3^ copies/mL	20	12	40.94	41.10	3208^*a*^	2906^*b*^
5 × 10^2^ copies/mL	20	2	41.05	41.05	2918^*c*^	2918^*d*^

a Mean Relative Fluorescence Unit value −34.12 for non-detections.

b Median Relative Fluorescence Unit value −43 for non-detections (range of values -115 to 120).

c Mean Relative Fluorescence Unit value −25.56 for non-detections.

d Median Relative Fluorescence Unit value −22.50 for non-detections (range of values −105 to 86).

### Utility of software-adjusted baseline correction slope limit in mitigation of false-positive Mgen MacR ASR results.

**FIG 2:**
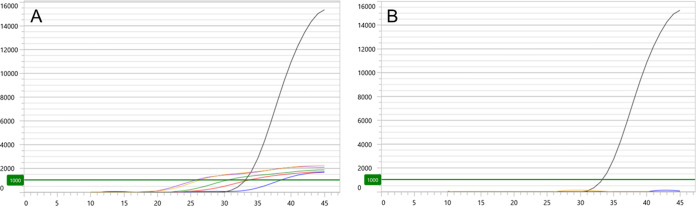
Mgen MacR ASR kinetic fluorescence output of experiments interrogating 3.3 × 10^9^ (purple lines), 10^9^ (yellow), 10^8^ (green), 10^7^ (red), and 10^5^ (blue) copies/mL of M. genitalium wild-type 23S rRNA template present in Aptima specimen tubes with default baseline correction slope limit of 50 units (A) and adjusted baseline correction slope limit of 250 units (B). Interrogations were made with PPR containing 0.6 μM primer sequence, 0.4 μM detection probe sequence, 2 mM MgCl_2_, and 60 mM KCl. *y* axis represents Relative Fluorescence Units (threshold set at 1000); *x* axis represents cycles of reverse transcriptase PCR. Wild-type kinetic data in both panels are compared to that of a known MRM RNA construct (black line).

With respect to M. genitalium wild-type RNA, beyond reduction of primer, detection probe, and MgCl_2_ (Table 1) concentrations, subsequent experiments revealed that rates of false-detection increased at higher template burden. When 60 mM KCl concentrations were utilized in PPR preparations, 100% of interrogations with 10^7^ copies/mL of wild-type RNA target yielded detectable signal (mean C*_T_* 33.62), 100% of interrogations with 10^8^ copies/mL yielded signal (mean C*_T_* 30.08), and 90% of interrogations with ≥ 10^9^ copies/mL of wild-type RNA yielded signal (mean C*_T_* 26.47). Kinetic data from these experiments demonstrated non-sigmoidal profiles (Fig. 2A).

By way of an adjustable baseline correction algorithm, MyAccess software had the capability of normalizing the slope of fluorescence kinetic profiles from samples known not to harbor MRM. When all collected wild-type data were re-analyzed by an adjusted baseline correction slope limit of 250 units, false-detections were not observed, regardless of input target concentration. Kinetic curves revealed negligible slope that did not approach the established fluorescence channel threshold (Fig. 2B). The baseline correction slope limit of 250 units therefore was applied for subsequent clinical specimen analysis.

### Performance of Mgen MacR ASR on primary clinical specimens initially testing positive for M. genitalium rRNA.

A total of 868 remnant primary clinical specimens with detectable M. genitalium rRNA were selected for assessment by Mgen MacR ASR (88.1% collected from males), following pretreatment protocols. Moreover, 304 specimens (35.0%) were derived from first-void urine (15.1% of urine specimens were derived from females), with the remainder consisting of swab collections from the oral cavity, rectum, and female lower reproductive tract. Within this M. genitalium-positive convenience sampling, 48 specimens yielded detectable C. trachomatis rRNA, 21 yielded detectable N. gonorrhoeae rRNA, and 1 yielded detectable T. vaginalis rRNA. Combinations of the aforementioned agents were detected in an additional 9 specimens.

Mgen MacR ASR internal control sequence was not detected in 2/868 specimens (0.23% non-valid rate within the convenience sample set). Of the remaining 866 positive specimens, 596 (68.8%) initially generated signal indicative of macrolide resistance (66.0% detection rate from female specimens, 69.2% from male specimens [*P = *0.51]; 63.9% detection rate from first-void urine specimens, 71.5% from swab specimens [*P = *0.02]) upon pretreatment with open access diluent additive and utilization of the default baseline correction slope limit. Within MRM specimens were 30 with detectable C. trachomatis rRNA, 11 with N. gonorrhoeae rRNA, 1 with T. vaginalis rRNA, and 3 with C. trachomatis plus N. gonorrhoeae or T. vaginalis rRNA.

**FIG 3:**
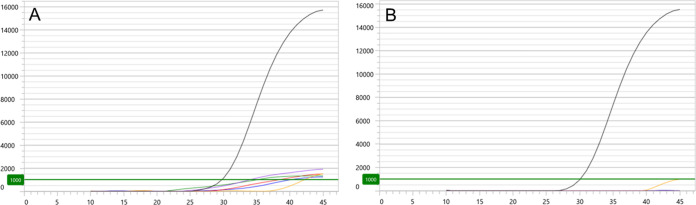
Kinetic fluorescence output of Mgen MacR ASR performed on 5 representative pretreated primary clinical swab specimens (purple, yellow, green, red, and blue lines) with default baseline correction slope limit of 50 units (A) and adjusted baseline correction slope limit of 250 units (B). Interrogations were made with PPR containing 0.6 μM primer sequence, 0.4 μM detection probe sequence, 2 mM MgCl_2_, and 60 mM KCl. *y* axis represents Relative Fluorescence Units (threshold set at 1000); *x* axis represents cycles of reverse transcriptase PCR. Kinetic data in both panels are compared to that of a known specimen positive for M. genitalium MRM (black line).

When an adjusted baseline correction slope limit of 250 units was applied to analysis of clinical specimens with detectable signal, interpretation of 13 specimens (11 swabs; 2 urines) reverted to negative (Fig. 3). This reduced final detection rates for urine specimens to 63.3%, swab specimens to 69.5% (*P = *0.06 versus urine specimens), male specimens to 67.5% (*P = *0.76 versus female specimens), and overall detection to 67.3% (583/866). Mean C*_T_* for data analyzed at the default baseline correction slope limit of 50 units and at the adjusted baseline correction slope limit of 250 units did not vary (*P = *0.23) (Table 4).

**TABLE 4:** Mean and median cycle threshold comparison of Mgen MacR ASR between pre-treated and untreated specimens, utilizing PPR of 0.6 μM primer sequence, 0.4 μM detection probe sequence, 2.0 mM MgCl_2_, 60 mM KCl, and baseline correction slope limit of 250 units as a reference method

Parameter	Analysis			
	Pre-treated specimens; default baseline correction slope limit of 50 units	Pre-treated specimens; adjusted baseline correction slope limit of 250 units (reference)		Untreated specimens; adjusted baseline correction slope limit of 250 units
Number of comparisons	583	583	528	528
Mean C*_T_*	33.81^*a*^	34.14	33.95	33.52^*b*^
Median C*_T_*	34.3	34.7	34.5	33.85

a *P* = 0.23 versus reference method.

b *P* = 0.14 versus reference method.

### Specificity assessments of Mgen MacR ASR.

Mock experimentation in which ~ 10^7^ organism inocula of macrolide-resistant S. pneumoniae or S. aureus were delivered to specimen transport tubes (*n* = 10 total) generated no positive results from Mgen MacR ASR (data not illustrated). Internal control signal was observed from each of these tests.

A convenience sampling of 141 primary clinical specimens yielding a negative result by the Aptima M. genitalium assay (72 urine specimens and 69 swab specimens) was subjected to Mgen MacR ASR following pretreatment protocols. Within these specimens, 33 (23.4%) yielded detectable C. trachomatis, N. gonorrhoeae, and/or T. vaginalis rRNA. All M. genitalium-negative specimens produced internal control-specific fluorescence (final non-valid rate 0.20%) but no detectable MRM signal in the Mgen MacR ASR after pretreatment with the open access diluent additive and subsequent analysis using default and adjusted baseline correction slope limit parameters.

### Mgen MacR ASR inhibition/interference assessments.

Primary urine (*n* = 40) and swab (*n* = 22) specimens with detectable MRM were treated with potentially inhibitory agents, then reanalyzed by Mgen MacR ASR following appropriate pretreatment protocols. Addition of gross blood or seminal fluid (10% final vol/vol for each) to primary specimens resulted in MRM detection rates of 93.3% and 93.8%, respectively (data not illustrated). One specimen incubated with gross blood that generated an MRM-negative result did generate internal control fluorescence. All MRM-positive specimens treated with gynecologic lubricant or talcum powder yielded a subsequent positive MRM result.

Residual MRM-positive primary swab specimens and primary urine specimens generated an expected MRM-positive result upon exposure to additive urine and rectal swab matrices, respectively, in 85.7% and 87.5% of instances. With respect to the discordant results, it is unclear if the result reversion was a by-product of inhibition or specimen dilution during experimentation. In total, 58/62 (93.5%) inhibition/interference assessments of previously-tested specimens yielded the expected MRM result.

### Assessment of Mgen MacR ASR in primary clinical specimens without pretreatment.

Following the initial primary clinical specimen investigation involving pretreatment, 817 M. genitalium-positive specimens contained sufficient quantity to allow for direct (without pretreatment) interrogation with optimized PPR preparations and baseline correction slope limit of 250 units. In 21 (2.6%) specimens, the pretreated specimen (reference) yielded detectable signal while the untreated specimen was negative. A total of 37 (4.5%) untreated specimens yielded a positive result when the reference method was negative (*P = *0.03 versus pretreated). Cumulative mean and median C*_T_* values for these 58 specimens were 40.3 and 40.4, respectively (data not illustrated). Within 528 specimens exhibiting concordant positive results between the pretreatment/untreated methods, mean C*_T_* did not vary (*P = *0.14; Table 4).

### Confirmation of MRM in primary clinical specimens by nucleic acid sequencing.

Of 95 untreated primary clinical specimens submitted for confirmatory Sanger sequencing, 88 (92.6%) yielded sufficient nucleic acid for analysis. One recent publication (21) reported a similar 89.4% rate of valid sequencing result using the same methodology. The 7 specimens failing to yield sufficient targets (including 3 first-void urine specimens) generated Mgen MacR ASR C*_T_* values ranging from 38.1 to 42.2 (median 40.2, mean 40.1; data not illustrated).

Within the 88 specimens yielding sufficient nucleic acid, overall agreement was 90.9%. These included 22 specimens in which MRM was not detected by Mgen MacR ASR and no MRM was detected by Sanger sequencing (100% negative agreement). Within the remaining 66 evaluable specimens, 58 demonstrated MRM by Sanger sequencing (87.9% positive agreement); corresponding median and mean Mgen MacR ASR C*_T_* values were 32.3 and 32.6, respectively. Included within the 58 Sanger sequencing MRM detections were 4 specimens yielding Mgen MacR ASR C*_T_* values of 40.9, 41.1, 42.0, and 42.7 (data not illustrated). Of the 8 evaluable specimens that did not demonstrate MRM by Sanger sequencing, 7 were first-void urine specimens that generated both median and mean Mgen MacR ASR C*_T_* values of 38.3.

## DISCUSSION

Analysis of MRM within M. genitalium-positive primary clinical specimens has, in early reports, achieved approximate detection rates of 40 to 60% in western Europe (31, 35, 36), 45 to 60% in Canada (37, 38), and 36% in Australia (19). A later Australian report (39) documented 62% MRM detection within 447 patients initially testing positive for M. genitalium. These data may signify recent increasing resistance, yet geographic distribution of patients cannot be discounted as a potential explanation for these differences. Nucleic acid sequencing is established as the reference method for making these and analogous determinations but can be limited by cost, throughput, and paucity of automation (40). Therefore, a commercial means of procuring M. genitalium MRM data relative to a primary clinical specimen would be advantageous and further assist providers in initial therapeutic intervention (24).

The Panther Fusion system open access channel has previously been utilized in direct detection or characterization of bacterial (41), parasitic (41), and viral (42–46) agents of disease and was posited in this study as an automated option for MRM determination within primary specimens testing positive for M. genitalium rRNA. Initial experimentation revealed that incubation of IVT wild-type 23S rRNA with high-concentration MRM primer and detection probe sequences, as well as an increased concentration of MgCl_2_, resulted in a propensity toward false-detections of macrolide resistance.

However, subsequent optimization of PPR stoichiometry by the laboratory and utilization of baseline slope correction by the laboratory effectively eliminated false-detections of macrolide resistance during interrogations of wild-type 23S rRNA template. Preliminary mitigation of false-detections occurred through reductions in MRM primer/detection probe and MgCl_2_ concentrations. Robustness of the assay was further demonstrated by experiments adjusting PPR KCl concentration using M. genitalium MRM target RNA. The ultimate means of mitigating the potential for false-detections within non-MRM template 23S rRNA was through use of an adjusted baseline correction slope limit of 250 units. This parameter, which can be programmed by a user into a Panther Fusion system open access protocol, did not result in significant changes in C*_T_* of clinical specimens demonstrating MRM (Table 4).

Upon optimization of PPR reagents, later experiments assessed lower limit of detection. While data consistently demonstrated M. genitalium MRM detection when introduced to Aptima specimen tubes at levels of ≥ 5 × 10^3^ copies/mL, challenges of 10^3^ copies/mL and 5 × 10^2^ copies/mL delivered to specimen tubes did result in limited detection. For example, the 60% rate of detection following 10^3^ copies/mL challenges yielded mean C*_T_*, median C*_T_*, and mean Relative Fluorescence Unit values of 40.94, 41.10, and 3208 units, respectively, for positive tests (Table 3). As reference, the mean Relative Fluorescence Unit value for negative tests was reported at -34.12. When the processing logistics of the Panther Fusion system are considered, limit of detection can further be expressed in terms of copies/reverse transcriptase PCR reaction. As one example, from Aptima specimen tubes containing 10^3^ copies/mL of template, 360 μL of volume is sampled by the Panther analyzer (theoretically removing 3.6 × 10^2^ copies per extraction). Extractions are then eluted within the Fusion analyzer using 50 μL of buffer, 5 μL of which (or 1/10) is utilized for reverse transcriptase PCR. Assuming 100% efficiency of purification of nucleic acid and that all 3.6 × 10^2^ copies are retained in the elution, 3.6 × 10^1^ copies are interrogated in the reverse transcriptase PCR reaction.

When a remnant M. genitalium-positive first-void urine specimen was subjected to Mgen MacR ASR at serial 10-fold dilutions, signal was detected at the 1:10 through 1:10,000 dilutions. Visual assessment of kinetic output confirmed a logical, stepwise shift in sigmoidal profiles throughout the dilution series (Fig. 1B). Of note, the C*_T_* for the specimen diluted 1:10,000 was 42.3. In additional experiments, Mgen MacR ASR-based detection of MRM was confirmed by Sanger sequencing from 4 primary clinical specimens that exhibited Mgen MacR ASR C*_T_* values ranging from 40.9 to 42.7. Taken together, these mock and clinical data suggest that the Mgen MacR ASR is a robust assay for detection of M. genitalium MRM, potentially including those that are manifested by C*_T_* values greater than 40. As a component of ASR or a laboratory-developed test development/verification, individual laboratories must verify such findings in their own setting.

In the Mgen MacR ASR, direct tube sampling occurs in the Panther analyzer, similar to the Aptima M. genitalium Assay. Following magnetic bead-assisted nucleic acid extraction in the Panther analyzer, extracts are transferred to the Fusion analyzer for real-time reverse transcriptase PCR and final completion of testing within 2 h and 30 min. Prior to specimen loading onto the Panther analyzer for extraction, manufacturer-suggested protocol (25) outlines pretreatment of primary clinical specimens with an open access diluent additive, a proprietary reagent purported to assist in the nucleic acid extraction process. Beyond hands-on technologist time being added to final turnaround time on account of this procedure, pretreatment of swab specimens with 300 μL of additive essentially creates a 1:2 dilution of the primary clinical specimen and, thus, may compromise assay sensitivity. Within a set of concordant pretreatment (reference method)-positive/untreated (undiluted)-positive specimens (*n* = 528), the mean C*_T_* from untreated specimens was lower than that from pretreatment specimens (Table 4), but this difference was not significant (*P = *0.14).

However, in an assessment of 58 M. genitalium-positive primary clinical specimens in which discordant pretreatment/untreated Mgen MacR ASR data were generated, the frequency of pretreatment-positive/untreated-negative Mgen MacR ASR results was decreased versus that of discordant pretreatment-negative/untreated-positive Mgen MacR ASR results (*P = *0.03). It should be noted that these discrepancies were typically found in specimens with low MRM burden (mean C*_T_* for discordant specimens yielding a positive pretreatment result was 40.0, analogous value for discordant specimens generating a positive untreated result was 40.6; data not illustrated). These data may provide additional considerations for laboratories during self-verification of the Mgen MacR ASR. Verification of processing protocols that do not call for pretreatment may expediently facilitate reflex Mgen MacR ASR without removing the primary clinical specimen from the Panther analyzer.

Our data reveal no significant differences in MRM detection when stratified by specimen source (urine versus swab *P = *0.06) and gender (*P = *0.76). An inherent limitation of convenience sampling may affect conclusions drawn from these data. Previous studies imply that M. genitalium macrolide resistance can be multifactorial in nature (19, 39). These findings do warrant additional epidemiologic studies of M. genitalium macrolide resistance using Mgen Mac ASR. Moreover, the convenience sampling method that was employed in the Mgen Mac ASR clinical assessment may not lead to completely accurate assessments of clinical sensitivity and specificity.

Positive and negative agreement values of 87.9% and 100%, respectively, were noted in a subset of specimens that were forwarded for Sanger sequencing. First-void urine specimens were largely contributory to the lower value of positive percentage agreement when comparing Mgen MacR ASR and Sanger sequencing results. At least 2 recent studies (47, 48) have described decreased Sanger sequencing MRM detection rates from primary urine specimens when compared to swab specimens. One publication inferred that overall first-void urine-based Sanger sequencing MRM detection rates within a testing population may be predicated by analytical sensitivity of the initial M. genitalium screening assay (48). In self-verification studies, laboratories may alternatively consider the value of pretreatment of first-void urine specimens prior to Mgen MacR ASR, as this protocol may contribute to greater rates of MRM detection when compared to Sanger sequencing.

In summary, performance of the commercial Mgen MacR ASR was optimized by PPR containing 0.6 μM M. genitalium macrolide resistance primer/0.4 μM M. genitalium macrolide resistance detection probe sequences, 0.6 μM internal control primer/0.4 μM internal control detection probe sequences, 2 mM MgCl_2_, and 60 mM KCl. These PPR findings were in conjunction with an adjusted baseline correction slope limit of 250 units to mitigate false-positive detections. This automated assay can facilitate appropriate therapeutic intervention for M. genitalium infection on the basis of the initial primary clinical specimen. These parameters can serve as a baseline for laboratories in verification of the assay in their own setting.
